# Variant level heritability estimates of type 2 diabetes in African Americans

**DOI:** 10.1038/s41598-024-64711-3

**Published:** 2024-06-18

**Authors:** Nicole D. Armstrong, Amit Patki, Vinodh Srinivasasainagendra, Tian Ge, Leslie A. Lange, Leah Kottyan, Bahram Namjou, Amy S. Shah, Laura J. Rasmussen-Torvik, Gail P. Jarvik, James B. Meigs, Elizabeth W. Karlson, Nita A. Limdi, Marguerite R. Irvin, Hemant K. Tiwari

**Affiliations:** 1 Department of Epidemiology, University of Alabama at Birmingham, Birmingham, AL USA; 2 Department of Biostatistics, University of Alabama at Birmingham, Birmingham, AL USA; 3 Center for Genomic Medicine, Massachusetts General Hospital, Boston, MA USA; 4 Department of Psychiatry, Massachusetts General Hospital, Boston, MA USA; 5 Division of Biomedical Informatics and Personalized Medicine, Department of Medicine, University of Colorado Anschutz Medical Campus, Aurora, CO USA; 6 Center for Autoimmune Genomics and Etiology, Cincinnati Children’s Hospital Medical Center, Cincinnati, OH USA; 7 Department of Pediatrics, Cincinnati Children’s Hospital Medical Center &, The University of Cincinnati, Cincinnati, OH USA; 8 Department of Preventive Medicine, Feinberg School of Medicine, Northwestern University, Chicago, IL USA; 9 Division of Medical Genetics, Department of Medicine, University of Washington, Seattle, WA USA; 10 Division of General Internal Medicine, Department of Medicine, Massachusetts General Hospital, Boston, MA USA; 11 Department of Medicine, Harvard Medical School, Boston, MA USA; 12 Program in Medical and Population Genetics, Broad Institute, Cambridge, MA USA; 13 Department of Medicine, Brigham and Women’s Hospital, Boston, MA USA; 14 Mass General Brigham Personalized Medicine, Boston, MA USA; 15 Department of Neurology, University of Alabama at Birmingham, Birmingham, AL USA

**Keywords:** Heritable quantitative trait, Type 2 diabetes mellitus, Genomics, Genetic polymorphisms, Disparities, Heritable quantitative trait, Type 2 diabetes, Genetics research, Medical ethics

## Abstract

Type 2 diabetes (T2D) is caused by both genetic and environmental factors and is associated with an increased risk of cardiorenal complications and mortality. Though disproportionately affected by the condition, African Americans (AA) are largely underrepresented in genetic studies of T2D, and few estimates of heritability have been calculated in this race group. Using genome-wide association study (GWAS) data paired with phenotypic data from ~ 19,300 AA participants of the Reasons for Geographic and Racial Differences in Stroke (REGARDS) study, Genetics of Hypertension Associated Treatments (GenHAT) study, and the Electronic Medical Records and Genomics (eMERGE) network, we estimated narrow-sense heritability using two methods: Linkage-Disequilibrium Adjusted Kinships (LDAK) and Genome-Wide Complex Trait Analysis (GCTA). Study-level heritability estimates adjusting for age, sex, and genetic ancestry ranged from 18% to 34% across both methods. Overall, the current study narrows the expected range for T2D heritability in this race group compared to prior estimates, while providing new insight into the genetic basis of T2D in AAs for ongoing genetic discovery efforts.

## Introduction

Diabetes mellitus is a heterogeneous group of metabolic and health conditions characterized by glucose dysregulation and defects in insulin secretion and/or insulin action^1^. Chronic hyperglycemia has been associated with long-term damage and dysfunction of the kidneys, heart, and blood vessels^2^. Diabetes is a major risk factor for cardiovascular disease (CVD), particularly coronary heart disease and stroke^3^. In the United States, type 2 diabetes (T2D) is the most common form of diabetes in adults, constituting > 90% of cases^4^ and is growing in prevalence among adolescents^5^. T2D is more often associated with increased age^6^; however, the T2D epidemic can largely be attributed to a worldwide increase in obesity^7^. Since lifestyle intervention focused on obesogenic behaviors is effective at preventing T2D^8^, identifying populations with increased genetic susceptibility could lower disease morbidity.

It is well-established that T2D is a complex disease, and the risk of developing T2D depends on environmental and genetic factors. Further supporting the genetic background of the disease is the high concordance in monozygotic twins compared to dizygotic twins in familial studies^9–12^. While traditional twin studies have been considered the “gold standard” for measuring the heritability of a trait, more recent literature has suggested that twin studies may overestimate heritability due to shared environment and non-additive genetic effects creating “phantom heritability”^13^.

While broad sense heritability of a trait is the proportion of phenotypic variation attributed to genetics, the narrow-sense heritability (h^2^) is the proportion attributable to the additive gene effects^14^. These additive effects of variants underlying a trait is of particular importance because they constitute the genetic variation component transferred from parent to offspring ^15^. With advances in genotyping technologies, the generation of genome-wide common genetic variant data has enabled approaches that provide an alternative to twin or family heritability studies^16,17^. These methods estimate heritability in unrelated individuals via correlation between genetic and phenotypic sharing, similar to family-based studies. However, instead of using the theoretic estimates of genetic sharing (i.e., Mendel’s Laws), single nucleotide polymorphism (SNP)-based heritability analyses allow for empirical estimates of genetic sharing to be directly obtained from the genotype data^17^. Thus, by extending the models to utilize the contributions from all common genetic variants, these methods can detect considerable shares of the additive genetic effect^13^.

While previous h^2^ estimates of T2D and related clinical traits (e.g., fasting glucose, fasting insulin) have varied between 25% and 80%, they have either excluded or underrepresented individuals of non-European ancestry, particularly African Americans (AAs)^2,18–22^. Importantly, known disparities exist in T2D, where AAs have a higher prevalence of T2D, increased mortality rates, and an increased risk of T2D complications compared to individuals of European descent^23,24^. Further, there is a concern that AAs and African ancestry groups may benefit less from genetic research, potentially exacerbating health disparities of common chronic conditions, such as T2D^25^. Given these trends, additional heritability studies are warranted for these populations. For example, accurate heritability estimates are needed to understand the utility of polygenic risk scores (PRS) in multi-ancestral populations with regard to the maximum trait variance expected to be explained by a PRS.

In the present study, we seek to capture the T2D additive genetic variation (i.e., h^2^) from ~ 19,300 AA participants in the Reasons for Geographic and Racial Differences in Stroke (REGARDS) study, the Genetics of Hypertension Associated Treatments (GenHAT) study, and the Electronic Medical Records and Genomics (eMERGE) network. Each study used an overlapping subset of 8.2 million imputed genetic variants to estimate the h^2^ using two common approaches, Genome-Wide Complex Trait Analysis (GCTA) and Linkage-Disequilibrium Adjusted Kinships (LDAK), making this one of the most extensive studies to estimate T2D heritability in AAs.

## Results

Descriptive statistics for 7957 AA T2D cases and 11,378 AA controls are presented in Table 1. On average, cases were slightly older (65 years versus 63 years for controls, 66 years versus 66 years for controls, and 68 years versus 67 years in controls for REGARDS, GenHAT, and eMERGE, respectively). Furthermore, T2D cases were more likely to be men in REGARDS (59%), but women in GenHAT (61%) and eMERGE (65%).

**Table 1:** Study level descriptive statistics.

	REGARDS		GenHAT		eMERGE	
	Cases	Controls	Cases	Controls	Cases	Controls
N	2516	5996	2776	2722	2665	2660
Age (years), mean ± SD (range)	64.54 ± 8.65 (45–92)	63.23 ± 9.38 (45–96)	65.97 ± 7.32 (55–92)	66.21 ± 7.71 (55–94)	67.57 ± 12.59 (31–99)	67.32 ± 12.88 (31–100)
Sex						
Female	41.02% (1,032)	38.41% (2,303)	60.66% (1,684)	49.85% (1,357)	64.77% (1,726)	66.24% (1,762)
Male	58.98% (1,484)	61.59% (3,693)	39.34% (1,092)	50.15% (1,365)	35.23% (939)	33.76% (898)

Study-level heritability estimates are provided in Table 2. The base model (Model 1) h^2^ estimates using LDAK were 19% in REGARDS, 19% in GenHAT, and 33% in eMERGE. Similar trends were observed when estimates were calculated using GCTA, ranging from 21% in GenHAT to 31% in eMERGE. Upon age, sex, and genetic ancestry adjustment (Model 3), h^2^ estimates from LDAK were similar to Model 1 (18% in GenHAT, 18% in REGARDS, and 33% for eMERGE). In the fully-adjusted (Model 3) using GCTA, h^2^ estimates for REGARDS, GenHAT, and eMERGE were 24%, 21%, and 32%, respectively. In a sensitivity analysis, the study site was included as a covariate for the eMERGE cohort and results remained similar with and without adjustment. Further, when adjusting for the AA-specific population prevalence of T2D (heritability liability, h^2^_liab_), estimates increased marginally across all models and methods (Supplemental Table 1).

**Table 2:** Study-level heritability (h2) estimates for T2D among 8,240,835 overlapping variants.

	**T2D Cases/Controls**	**Case proportion**	**Method**	**Model 1**	**Model 2**	**Model 3**
				**h**^**2**^** (SE)**	**h**^**2**^** (SE)**	**h**^**2**^** (SE)**
**REGARDS**	2516/5996	0.30	LDAK	0.19 (0.05)	0.18 (0.05)	0.18 (0.05)
			GCTA	0.25 (0.06)	0.24 (0.06)	0.24 (0.06)
**GenHAT**	2776/2722	0.50	LDAK	0.19 (0.08)	0.19 (0.08)	0.18 (0.08)
			GCTA	0.21 (0.10)	0.21 (0.10)	0.21 (0.10)
**eMERGE**	2665/2660	0.50	LDAK	0.33 (0.08)	0.33 (0.08)	0.33 (0.08)
			GCTA	0.31 (0.09)	0.32 (0.09)	0.32 (0.09)

*SE* standard error.

Model 1: Base model.

Model 2: PC 1–4.

Model 3: PC 1–4 + age + sex.

## Discussion

African American populations continue to be underrepresented in genomic research. In the era of genomic medicine, this could unintentionally create new disparities in prevention and prediction of T2D. By leveraging genome-wide SNP array data from unrelated participants belonging to well-characterized cohort studies the current study provides insight into the additive genetic factors that contribute to the phenotypic variance of T2D in this non-European population. This type of common genetic variant heritability study is less prone to the confounding effects of shared environment observed in family studies. Therefore, the results presented may give a more precise estimation of the genetic contribution to T2D which may inform how genome-wide data is used in the future for the treatment and prevention of the condition.

We estimated the h^2^ of T2D using two well-characterized estimation approaches, LDAK and GCTA. Upon covariate adjustment, we observed h^2^ estimates from GCTA ranging from 21 to 32%. LDAK provided more conservative fully-adjusted estimates, ranging from 18 to 33%. This is most likely due to the inclusion of LD and allele frequency patterns into the estimation, correcting uneven LD patterns across the genome^26^. Subsequent h^2^_liab_ estimates, aimed to measure heritability independent of disease prevalence, ranged from 19% to 34%.

As described, we observed variable estimates across studies. This leads us to believe that age and sex were confounders, justifying the need for a fully adjusted model and age-matching cases and controls in eMERGE to be representative of what was present in GenHAT and REGARDS. The h^2^_liab_ estimates also showed slight inflation in REGARDS across both methods and we speculate that could be a result of the smaller case proportion (~ 30%) compared to eMERGE and GenHAT (~ 50%). It is also important to note that h^2^_liab_ could potentially be biased, as previously reported in Golan et al., as it is a function of the prevalence in a given sample population^27^.

Prior reports of heritability of T2D and T2D-related traits (ranging 25–80%) have been described largely in populations or families of European descent. A 2005 familial aggregation study in ~ 2400 non-diabetic AA participants, described heritability of 29% ± 9% for fasting glucose and 28% ± 8% for fasting insulin measures^19^, similar to our findings. Importantly, over 700 unique genetic loci associated with T2D have been reported by GWAS^28–32^, explaining almost 20% of T2D heritability in a multi-ancestry sample^31^. The majority of these loci were identified in European or Asian populations^33^, though > 25 loci were discovered in AAs^31,34–36^. Given the wide-range of reported heritability estimates it’s difficult to decipher how much missing heritability remains after accounting for discovered significant loci. Therefore, more precise estimates of heritability such as that presented in our study are needed to inform future GWAS discovery and precision medicine applications of GWAS data.

Our study is subjected to several limitations. First, heterogeneity in the estimates could result from heterogeneity in the T2D phenotype, heterogeneity in study designs, and population admixture. To account for these limitations, we adjusted for genetic principal components in each study, as well as the study site in sensitivity models in eMERGE. We followed the same validated T2D phenotyping as a previously published report^37^, attempting to harmonize the phenotype between the population-based longitudinal study (REGARDS), randomized clinical trial (GenHAT/ALLHAT), and electronic medical records (EMR) from eMERGE. While attempting to harmonize the genetic variants, it is important to note the differences in the imputation reference panel between REGARDS and GenHAT (TOPMed release 2) versus eMERGE (HRC), which did result in the exclusion of nearly 7 million variants from REGARDS and GenHAT. However, this number of variants is still compatible with other publications that have used these approaches^38^. Lastly, while the use of the contemporary arrays and methodologies (e.g., Illumina MEGA array and TOPMed reference panel) allows for interrogation of more African-specific variants, the potential genetic contribution of rare minor allele frequency (MAF) < 1% or structural variants was not investigated in this study. This is important since rare variants are thought to harbor more deleterious effects than common variants, as well as playing an important role in accurately calculating the heritability of complex diseases^39^.

In conclusion, we conducted one of the largest heritability estimates of T2D to date utilizing genetic array data and to the best of our knowledge, the largest study of h^2^ in AAs, comprising three independent studies with sizable AA populations and T2D prevalence. All three studies have well-described and extensive phenotyping, allowing for appropriate covariate adjustment and previously validated T2D case and control definitions. The use of imputed data allows for more consistency across studies, with the inclusion of more than 8.2 million overlapping genetic variants. Though we observed similar trends using LDAK and GCTA, the considerations of LD resulted in a more conservative h^2^ range across studies using LDAK. Our h^2^ estimates are lower with smaller confidence intervals than most familial studies on T2D that have been predominately described in European populations; however, they are similar to a family study on fasting insulin and glucose levels in AAs. Future work in determining the heritability of complex diseases, including T2D, is warranted in order to advance the availability of genomic resources for non-European populations.

## Methods

### Study populations

**Figure 1:**
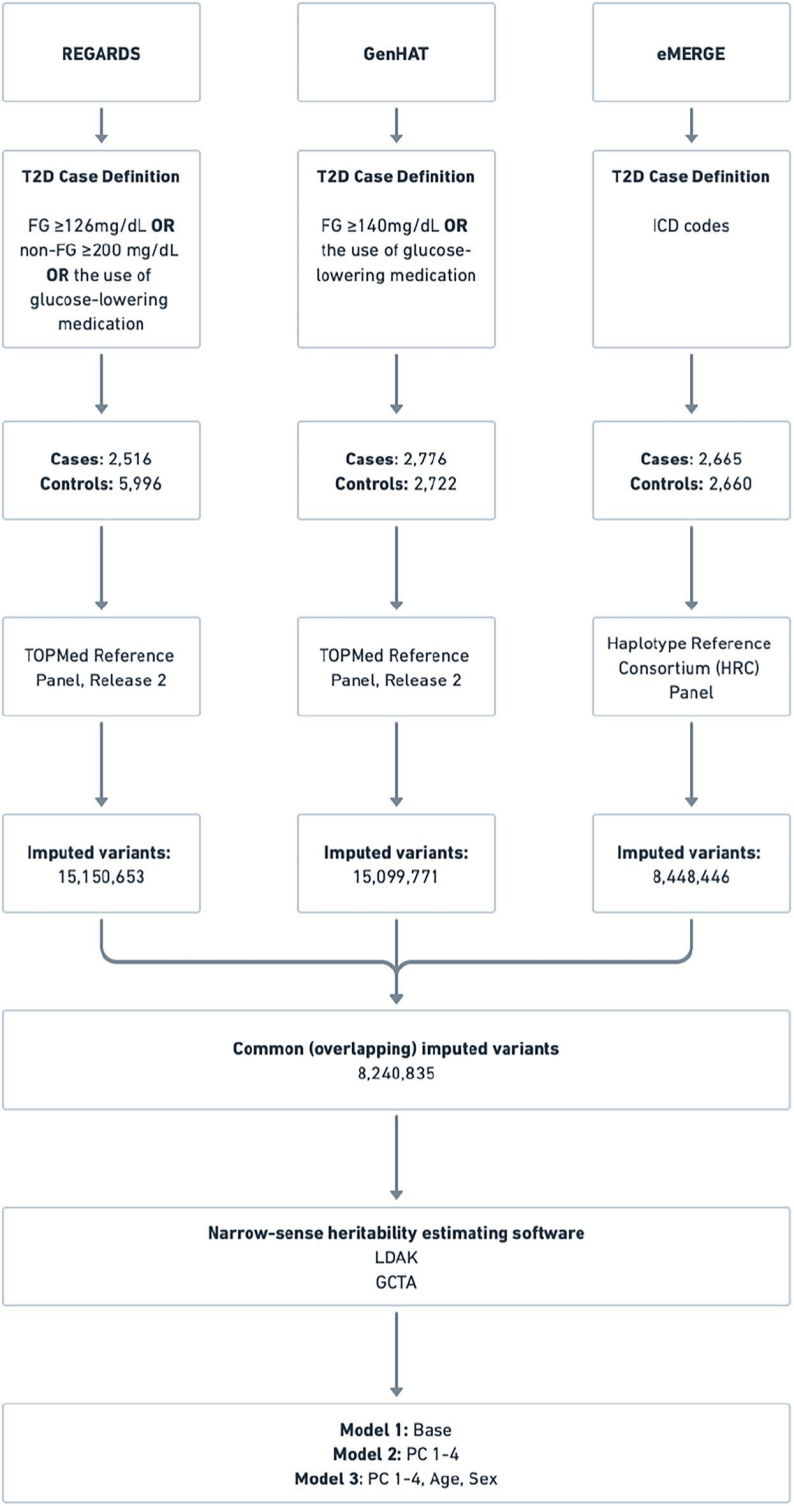
Study Overview. *T2D* type 2 diabetes, *FG* fasting glucose, *ICD* International Classification of Diseases, *GCTA* genome-wide complex trait analysis, *LDAK* linkage disequilibrium adjusted kinships, *PC* principal components.

Three cohorts, REGARDS, GenHAT, and eMERGE contributed genetic data for this study, comprising 7,957 T2D cases and 11,378 controls of African descent (Fig. 1). Descriptive statistics for each study are described in Table 1. Signed informed consent was collected from all participants in each study. All studies were reviewed and approved by the institutional review boards of the participating institutions and study sites.

### Reasons for geographic and racial differences in stroke (REGARDS) study

REGARDS is a national, longitudinal study of incident stroke and associated risk factors, enrolling over 30,000 Black and white adults aged 45 years or older from all 48 contiguous US states and the District of Columbia^40^. Participants completed a computer-assisted telephone interview (CATI) and an in-home visit where blood and urine were collected, as well as blood pressure measurements and a medicine review. Participants are contacted at 6 month intervals to obtain information regarding incident stroke or secondary outcomes. A subset of 8916 Black REGARDS participants underwent genotyping using Illumina Infinium AMR/AFR (MEGA) BeadChip arrays. Quality control procedures have been previously described^41^, but briefly, participants were excluded based on sex mismatches, internal duplicates, or HapMap controls. Variants were excluded if they were located on sex chromosomes, had ambiguous strands, were not bi-allelic, were in violation of Hardy Weinberg (p < 1.00e-12 for REGARDS), had MAF < 5%, and/or had a missing rate > 10%. Imputation was performed using the Trans-omics for Precision Medicine (TOPMed) release 2 (Freeze 8) reference panel^42^.

### Genetics of hypertension associated treatments (GenHAT) study

GenHAT is an ancillary study to the Antihypertensive and Lipid Lowering Treatment to Prevent Heart Attack Trial (ALLHAT). ALLHAT was a randomized, double-blind multicenter clinical trial that enrolled over 42,000 high-risk individuals 55 years or older with hypertension and at least one additional risk factor for cardiovascular disease^43,44^. The GenHAT study (N = 39,114) evaluated the interaction between candidate hypertensive genetic variants and antihypertensive treatments to modify the risk of CVD outcomes. A subset of 7711 Black adults with hypertension was genotyped on Illumina MEGA BeadChip arrays. Similar to GenHAT, QC procedures have been previously described^41^. Participants were excluded based on sex mismatches, internal duplicates, or HapMap controls, while variants were excluded if they were located on sex chromosomes, had ambiguous strands, were not bi-allelic, were in violation of Hardy Weinberg (p < 1.00e-05), had MAF < 5%, and/or had a missing rate > 10%. Imputation was performed using the Trans-omics for Precision Medicine (TOPMed) release 2 (Freeze 8) reference panel^42^.

### Electronic medical records and genomics (eMERGE) network

The eMERGE network combines DNA biorepositories with electronic medical records (EMR) for the purpose of research focused on advancing efforts in genomic medicine. The eMERGE III cohort was initiated in 2015 and culminated in over 100,000 GWAS samples across the network, while eMERGE IV aimed to develop and disseminate methodologies for genetic risk assessment, integrate genetics into routine medical practice to identify individuals at high risk for common disease, and recommend interventions^37,45^. For the current study, genetic data from eight sites (Cincinnati Children’s Hospital Medical Center, Children’s Hospital of Philadelphia, Columbia University, Mass General Brigham, Mayo Clinic, Icahn School of Medicine at Mount Sinai, Northwestern University, and Vanderbilt University Medical Center)^46^ was imputed against the Haplotype Reference Consortium (HRC) panel^47^.

To age-match cases and controls from eMERGE to the REGARDS and GenHAT studies, samples were selected from the age range 30–100. Individuals were divided into deciles and numbers of cases and controls were matched in each decile (Supplemental Fig. 1).

### Definition of type-2 diabetes status

T2D status was defined independently across all three studies. In the REGARDS study, T2D cases were classified based on fasting glucose ≥ 126 mg/dL (7 mmol/L), non-fasting glucose ≥ 200 mg/dL (11.1 mmol/L), or the use of diabetes medications (e.g., oral hypoglycemic pills or insulin). The ALLHAT/GenHAT definition of T2D was described as a fasting glucose ≥ 140 mg/dL or use of diabetes medication^43,44^. Therefore, in the current study we excluded controls that had baseline fasting glucose ≥ 126 mg/dL or missing a fasting glucose measure. In eMERGE, a revised EMR-based phenotyping algorithm based on ICD9/ICD10 codes was applied across the participants^37^. Further information regarding all T2D definitions has been previously described in detail^37^.

### Statistical analysis

To estimate the h^2^ for T2D, we employed two widely used methodologies, GCTA and LDAK, using an overlapping subset of 8,240,835 imputed genetic variants (Fig. 1). Three statistical models were fit: a base model without covariates (Model 1), a model adjusting for genetic ancestry through principal component analysis^48^ (Model 2), and a model adjusting for genetic ancestry, age, and sex (Model 3). In addition to the stated Model 3, a sensitivity analysis further adjusting for the study site to account for potential heterogeneity in sample characteristics across sites in the eMERGE cohort was performed, and the results remained consistent.

### Genome-wide complex trait analysis (GCTA) method

Both genotypes and imputed variants that passed quality control (QC) filters were used to construct a genomic relationship matrix (GRM) using the GCTA tool, as previously described using the genome-based restricted maximum likelihood (GREML)^49,50^. The GRM reflects allele sharing (${A}_{ij}$) between two individuals (*i* and *j*) across variants with entries

1 $$A_{ij} = \frac{1}{m}\mathop \sum \limits_{k = 1}^{k = m} \frac{{\left( {x_{ik} - 2p_{k} } \right)\left( {x_{jk} - 2p_{k} } \right)}}{{2p_{k} \left( {1 - p_{k} } \right)}},$$

where *m* is the number of variants, *x*_*ik*_ and *x*_*jk*_ are the genotypes coded as 0, 1, or 2 of individuals i and *j,* respectively, at the *k*^*th*^ locus, and *p*_*k*_ is the MAF of the *k*^*th*^ locus. The variance of T2D was calculated as

2 $$var\left( {T2D} \right) = A\sigma_{v}^{2} + I\sigma_{e}^{2} ,$$

where the variance explained by the genetic variants (${\sigma }_{v}^{2}$) corresponding to GRM and residual error variance (${\sigma }_{e}^{2}$) were estimated using restricted maximum likelihood (REML), *A* is an *n* × *n* matrix with elements *A*_*ij,*_ and I is an *n* × *n* identity matrix. The proportion of the variance of T2D explained by all the genetic variants (h^2^) on the observed scale was then calculated as:

3 $$h^{2} = \frac{{\sigma_{v}^{2} }}{{\left( {\sigma_{v}^{2} + \sigma_{e}^{2} } \right)}}$$

We removed one individual from relative pairs with estimated genetic relatedness greater than 0.025 to ensure no closely-related individuals were included in heritability estimates (e.g., parent-offspring, siblings, cousins).

### Linkage-disequilibrium adjusted kinships (LDAK) method

Knowing that African ancestral populations have greater haplotype diversity and, in turn, shorter segments of linked alleles^51^, we utilized LDAK, which incorporates linkage disequilibrium (LD), as an additional approach. LDAK can be used as an alternative method of generating a GRM by weighting genetic variants based on local LD patterns^52^. As previously described, the genetic variance of variants in high LD with a causal variant is typically overestimated in GCTA, while the genetic variance is underestimated in lower LD regions^52^, thus demonstrating the importance of accounting for LD in the construction of the LD-weighted GRM. Therefore LD-weighting eliminates the overestimation of heritability in high LD regions and underestimation of heritability in low LD regions by giving smaller weights to markers in the high-LD regions and large weights to markers in low LD regions^53^.

The GRM for LDAK is constructed as follows:

4 $$GRM_{LDAK} = \frac{XWX^{\prime}}{m}$$

where W is the diagonal matrix with elements representing the LD-weight for each variant, N is the total number of variants, and X is a matrix with the general term

5 $$x_{ij} = \left( {m_{ij} - 2_{pj} } \right) / \left( {\sqrt {2_{pj} \left( {1 - p_{j} } \right)} } \right)$$

with ${p}_{j}$ being the frequency of a given allele at variant *j* and ${m}_{ij}$ being the genotype for the *j-th* variant in the *i-th* individual (represented by 0, 1, or 2).

When estimating heritability, LDAK assumes:

6 $$E\left[ {h_{j}^{2} } \right] \propto \left[ {f_{i} \left( {1 - f_{i} } \right)} \right]^{1 + \alpha } \times \varpi_{j} \times r_{j}$$

where $E\left[{h}_{j}^{2}\right]$ is the expected heritability contribution of genetic variant *j* and ${f}_{i}$ is its observed MAF. The parameter α determines the assumed relationship between heritability and MAF. The genetic variant weighs ( ${\varpi }_{j}$) are based on the local level of LD and tend to be higher for variants in low-LD regions; thus, LDAK assumes that these variants contribute more than those in the high-LD areas. ${r}_{j}$ $\epsilon \left[\text{0,1}\right]$ is an information score measuring genotype certainty, where LDAK assumes higher-quality variants contribute more than lower-quality ones^54^.

### Liability scale

In order to account for the inflated proportion of cases in case–control designs, the heritability estimation on the observed scale was transformed to that on the liability as

7 $$h_{liab}^{2} = h^{2} \frac{{K\left( {1 - K} \right)}}{{z^{2} }} \frac{{K\left( {1 - K} \right)}}{{P\left( {1 - P} \right)}},$$

where $K$ is the population prevalence of the T2D in AAs,* P* is the sample prevalence of T2D, and z is the height of the standard normal probability density function at the truncation threshold t, as previously described^55^. The AA-specific T2D prevalence of 12.5% was extracted from recent literature^56^.

### Supplementary Information

Supplementary Information.

## Data Availability

The REGARDS (Study Accession: phs002719.v1.p1), GenHAT (Study Accession: phs002716.v1.p1) and eMERGE (Study Accession: phs001584.v2.p2) phenotypic and genetic data are available on dbGaP.
