# Supplementary material for: Variant level heritability estimates of type 2 diabetes in African Americans
Source: Sci Rep. 2024 Jun 18;14:14009. doi: 10.1038/s41598-024-64711-3 (PMC11189523; doi:10.1038/s41598-024-64711-3)
Supplement: Supplementary file 1 — Supplementary Information. [file 41598_2024_64711_MOESM1_ESM.docx]

**Supplemental Material**

**Variant level heritability estimates of type 2 diabetes in African Americans**

**Nicole D. Armstrong**^1*^**, Amit Patki**^2^**, Vinodh** **Srinivasasainagendra**^2^**, Tian Ge**^3,4^**, Leslie A. Lange**^5^**, Leah Kottyan**^6^**, Bahram Namjou**^6^**, Amy S. Shah**^7^**, Laura J. Rasmussen-Torvik**^8^**, Gail P. Jarvik**^9^**, James B. Meigs**^10,11,12^**, Elizabeth W Karlson**^13,14^**, Nita A. Limdi**^15^**, Marguerite R. Irvin**^1^**, and Hemant K. Tiwari**^2^

^1^ Department of Epidemiology, University of Alabama at Birmingham, Birmingham, AL, USA

^2^ Department of Biostatistics, University of Alabama at Birmingham, Birmingham, AL, USA

^3^ Center for Genomic Medicine, Massachusetts General Hospital, Boston, MA, USA

^4^ Department of Psychiatry, Massachusetts General Hospital, Boston, MA, USA

^5^ Division of Biomedical Informatics and Personalized Medicine, Department of Medicine, University of Colorado Anschutz Medical Campus, Aurora, CO, USA

^6^ Center for Autoimmune Genomics and Etiology, Cincinnati Children’s Hospital Medical Center, Cincinnati, OH, USA

^7^ Department of Pediatrics, Cincinnati Children’s Hospital Medical Center & The University of Cincinnati, Cincinnati, OH, USA

^8^ Department of Preventive Medicine, Feinberg School of Medicine, Northwestern University, Chicago, IL, USA ^9^ Division of Medical Genetics, Department of Medicine, University of Washington, Seattle, WA, USA

^10^ Division of General Internal Medicine, Department of Medicine, Massachusetts General Hospital, Boston, MA, USA

^11^ Department of Medicine, Harvard Medical School, Boston, MA, USA
^12^ Program in Medical and Population Genetics, Broad Institute, Cambridge, MA, USA

^13^ Department of Medicine, Brigham and Women’s Hospital, Boston, MA, USA
^14^ Mass General Brigham Personalized Medicine, Boston, MA, USA

^15^ Department of Neurology, University of Alabama at Birmingham, Birmingham, AL, USA

*** Correspondence:**Corresponding Author: Nicole D. Armstrong
[nmda@uab.edu](mailto:nmda@uab.edu)

| **Supplemental Table 1: Narrow-sense heritability (h^2^) estimates for T2D using LDAK and GCTA with liability estimates (n=8,240,835 variants).** | | | | | | | | | |
| --- | --- | --- | --- | --- | --- | --- | --- | --- | --- |
|  | **T2D Cases/Controls** | **Case proportion** | **Method** | **Model 1** | | **Model 2** | | **Model 3** | |
|  |  |  |  | **h^2^ (SE)** | **h^2^_liab_** **(SE)^*^** | **h^2^ (SE)** | **h^2^_liab_ (SE)^*^** | **h^2^ (SE)** | **h^2^_liab_** **(SE) ^*^** |
| **REGARDS** | 2516/5996 | 0.30 | LDAK | 0.19 (0.05) | 0.23 (0.06) | 0.18 (0.05) | 0.22 (0.06) | 0.18 (0.05) | 0.22 (0.06) |
|  |  |  | GCTA | 0.25 (0.06) | 0.31 (0.07) | 0.24 (0.06) | 0.29 (0.07) | 0.24 (0.06) | 0.29 (0.07) |
| **GenHAT** | 2776/2722 | 0.50 | LDAK | 0.19 (0.08) | 0.20 (0.08) | 0.19 (0.08) | 0.20 (0.08) | 0.18 (0.08) | 0.19 (0.08) |
|  |  |  | GCTA | 0.21 (0.10) | 0.22 (0.10) | 0.21 (0.10) | 0.22 (0.10) | 0.21 (0.10) | 0.22 (0.10) |
| **eMERGE** | 2665/2660 | 0.50 | LDAK | 0.33 (0.08) | 0.34 (0.08) | 0.33 (0.08) | 0.34 (0.08) | 0.33 (0.08) | 0.34 (0.08) |
|  |  |  | GCTA | 0.31 (0.09) | 0.32 (0.09) | 0.32 (0.09) | 0.33 (0.10) | 0.32 (0.09) | 0.32 (0.10) |
| **Abbreviations**: SE- standard error  Model 1: Base model Model 2: PC 1-4 Model 3: PC 1-4 + age + sex  *AA-specific T2D population prevalence of 12.5% | | | | | | | | | |

**Supplemental Figure 1: eMERGE participants histogram.** Panel A.) eMERGE participant’s age distribution. Panel B.) Age-matched eMERGE participant’s age distribution. **T2D Cases**: diabetes=1; **Controls**: diabetes =0.
